# Methotrexate-Loaded Solid Lipid Nanoparticles: Protein Functionalization to Improve Brain Biodistribution

**DOI:** 10.3390/pharmaceutics11020065

**Published:** 2019-02-02

**Authors:** Elisabetta Muntoni, Katia Martina, Elisabetta Marini, Marta Giorgis, Loretta Lazzarato, Iris Chiara Salaroglio, Chiara Riganti, Michele Lanotte, Luigi Battaglia

**Affiliations:** 1 Dipartimento di Scienza e Tecnologia del Farmaco, Università degli Studi di Torino, 10124 Torino, Italy; elisabetta.muntoni@unito.it (E.M.); katia.martina@unito.it (K.M.); elisabetta.marini@unito.it (E.M.); marta.giorgis@unito.it (M.G.); loretta.lazzarato@unito.it (L.L.); 2 Dipartimento di Oncologia, Università degli Studi di Torino, 10043 Orbassano, Italy; irischiara.salaroglio@unito.it (I.C.S.); chiara.riganti@unito.it (C.R.); 3 Dipartimento di Neuroscienze, Università degli Studi di Torino, 10126 Torino, Italy; michele.lanotte@unito.it

**Keywords:** solid lipid nanoparticles, blood–brain barrier, methotrexate, insulin, transferrin

## Abstract

Glioblastoma is the most common and invasive primary tumor of the central nervous system and normally has a negative prognosis. Biodistribution in healthy animal models is an important preliminary study aimed at investigating the efficacy of chemotherapy, as it is mainly addressed towards residual cells after surgery in a region with an intact blood–brain barrier. Nanoparticles have emerged as versatile vectors that can overcome the blood–brain barrier. In this experimental work, solid lipid nanoparticles, prepared using fatty acid coacervation, have been loaded with an active lipophilic ester of cytotoxic drug methotrexate, and functionalized with either transferrin or insulin, two proteins whose receptors are abundantly expressed on the blood–brain barrier. Functionalization has been achieved by grafting a maleimide moiety onto the nanoparticle’s surface and exploiting its reactivity towards thiolated proteins. The nanoparticles have been tested in vitro on a blood–brain barrier cellular model and in vivo for biodistribution in Wistar rats. Drug metabolites, in particular 7-hydroxymethotrexate, have also been investigated in the animal model. The data obtained indicate that the functionalization of the nanoparticles improved their ability to overcome the blood–brain barrier when a PEG spacer between the proteins and the nanoparticle’s surface was used. This is probably because this method provided improved ligand–receptor interactions and selectivity for the target tissue.

## 1. Introduction

Glioblastoma multiforme (GB), a grade IV glioma, is the most frequently occurring and invasive primary tumor of the central nervous system (CNS) and causes approximately 4% of cancer-associated deaths, making it one of the most fatal cancers worldwide. Median survival with present therapies is about 14 months, and the 2-year survival rate is merely 3–5% [1].

GB pharmacological therapy is currently only adjuvant; it is used subsequent to surgery and either subsequent to or simultaneously with radiotherapy. It is rarely applied as the first treatment option. GB chemotherapeutic drugs are rarely effective despite the fact that the blood–tumor barrier (BTB), in malignant brain tumors, is more permeable than the blood–brain barrier (BBB) [2]. In fact, drugs should mainly be addressed towards the eradication of residual tumor cells after surgery or radiotherapy. This cell population includes cells that migrate from the tumor into healthy tissue (and which were not destroyed by previous treatment) or that feed the tumor from, still active, distant sites. The critical zone for the treatment of glioblastoma is the area surrounding it, named BAT (Brain Adjacent to Tumor), including cells in the invasion phase and those coming into the tumor, which, unlike those within the tumor, maintain an intact BBB. Since BAT is the main target of chemotherapy, the development of chemotherapeutic formulations that can cross an intact BBB is essential [3]. Classical cytotoxic drugs, which have high in vitro efficacy against GB, have difficulty in overcoming the BBB. The BBB is, therefore, a major limitation to drug therapy [4]. Moreover, in addition to tight junctions, the BBB also bears, on the luminal surface, many efflux membrane transporters, such as P-glycoprotein (Pgp) and multidrug-related proteins (MRP) that recognize several anticancer drugs as substrates [5]. The biodistribution of cytotoxic drugs in healthy animal models with intact BBB is, therefore, an important preliminary study that can be used to foresee the efficacy of chemotherapy against GB.

Nanocarriers are drug transport systems that have gained a great deal of attention over recent decades for their ability to facilitate site-specific drug delivery, including brain delivery [6]. In particular, solid lipid nanoparticles (SLNs) have been proposed as innovative vehicles for chemotherapeutic agents in experimental GB treatment because of their ability to enhance drug uptake by cells and to evade the Pgp efflux system. It is well known that SLNs are subject to endocytosis by endothelial cells and can be targeted to specific tissues [7].

In a recent work, SLNs were prepared using an innovative technology (coacervation method), and loaded with a lipophilic ester prodrug of methotrexate (MTX)—didodecylmethotrexate (ddMTX)—with good entrapment efficiency. ddMTX-loaded SLNs demonstrated promising cytotoxicity against GB primary cell cultures [8]. The subsequent in vivo biodistribution studies, performed in healthy rats and in glioma models, gave encouraging results. However, the ability to overcome the BBB was still too poor to achieve significant results on tumor growth in glioma models [9]. Major improvements would include an increase in the SLN plasmatic concentration, and the implementation of a successful BBB targeting strategy. To this aim, this experimental work improves the ddMTX-loaded SLN formulation technique by reducing the size and polydispersity of the nanoparticles and optimizing their hydrophilic coating. Moreover, ddMTX-loaded SLNs were functionalized with either transferrin (TRF) or insulin (INS), two proteins whose receptors are abundantly expressed on the BBB, using maleimide-thiol chemistry. The nanoparticles were tested in vitro on blood–brain barrier cell models and in vivo for biodistribution on Wistar rats. In vivo drug metabolism was also investigated in the animal model.

## 2. Materials and Methods 

### 2.1. Materials

Sodium behenate was from Nu-Chek Prep, Inc. (Eleisyan, MN, USA); sodium stearate, bovine INS, TRF, dimethylsulfoxide (DMSO), dimethylformammide (DMF), MTX, stearylamine, sodium dodecyl sulfate (SDS), potassium permanganate, hydrogen peroxide (H_2_O_2_), acetic acid, triethylamine, dextran (*M*_w_ 60,000–90,000), tris(carboxyethyl)phosphine hydrochloride (TCEP), iminothiolane, 3-maleimidobenzoic acid *N*-hydroxysuccinimide ester (MBS), *N*-hydroxysuccinimide (NHS), 1-ethyl-3-(3-dimethylaminopropyl)carbodiimide (EDC), 3-methylisoquinoline, ethanol, methanol, acetonitrile, hydrolyzed polyvinyl alcohol of 89% 80,000–120,000 *M*_w_ (PVA120000) and 80% hydrolyzed polyvinyl alcohol of 9000–10,000 *M*_w_ (PVA9000) were from Sigma Aldrich (Saint Louis, MO, USA); stearic acid, tris(hydroxymethyl)aminomethane (TRIS), phosphoric acid, hydrochloric acid, sodium acetate, ammonium chloride, potassium chloride, ammonium sulphate, potassium ferricyanide and sodium hydroxide were from Merck (Darmstadt, Germany); ethylendiaminotetraacetic acid (EDTA), chloroform, methanol and ethanol were from Carlo Erba (Cornaredo, Italy); diaminopoliethylenglycol 2000 *M*_w_ (diamino-PEG) was from Iris Biotech GmbH (Marktredwitz, Germany); lactic acid was from A.C.E.F. (Fiorenzuola, Italy); deionized water was obtained by a MilliQ system (Millipore, MO, USA). All other chemicals were of analytical grade and used without any further purification.

Electrophoresis reagents were from Bio-Rad Laboratories (Hercules, CA, USA). When not otherwise specified, all the other reagents were from Sigma Chemicals Co.

#### 2.1.1. Cells

Human Cerebral Microvessel Endothelial Cells/Dilution Cloning Number 3 (hCMEC/D3 cells), a human brain microvascular endothelial cell line that retains the properties of BBB in vitro, were cultured as reported [10,11]. Cells were seeded at 50,000/cm^2^ and grown for 7 days up to confluence in Petri dishes and Transwell devices (0.4 µm diameter pores-size, Corning Life Sciences, Chorges, France). 

#### 2.1.2. Animals

Male Wistar rats (Charles River, MA, USA), weighing 200–250 g, were housed in standard facilities, handled and maintained according to our Institutional Animal Care and Use Committee ethical regulations. The rats were kept under controlled environmental conditions (23 ± 1 °C, 50–60% humidity, 12 h light and dark cycles, lights on at 7:00 a.m.). The rats were given ad libitum access to food and water. The procedures conformed to the Ethics Committee of the University of Turin’s institutional guidelines on animal welfare (DL 26/2014 implementation of directive 2010/63 UE) as well as International Guidelines, and all efforts were done to minimize the number of animals and their discomfort. All experiments on animal models were performed according to an experimental protocol approved by the University Bioethical Committee and the Italian Ministry of Health (Prot. N. 135/2015, 29/09/2015).

### 2.2. Methods

#### 2.2.1. ddMTX Synthesis

The lipophilic ester, ddMTX, was synthesized according to the literature [12,13]. Briefly, 100 mg MTX and 71.5 mg cesium carbonate and 120 μL dodecyl bromide (corresponding to 125 mg; d = 1.04) were dissolved in 6 mL DMSO and left to react under stirring for 24 h. The molar ratio between MTX, cesium carbonate and dodecyl bromide is 1:1:2. ddMTX was precipitated from the reaction mixture by adding 5 mL water and then extracted with 3 mL chloroform, which was repeated 5 times. The organic phase was reduced to 6 mL under nitrogen steam, and DMSO was removed by extracting with 5 mL sodium chloride saturated solution, which was repeated 3 times. The organic phase was then evaporated under nitrogen steam and the residue was purified through Silica column by employing a chloroform/methanol gradient. 

The obtained product was characterized through High Performance Liquid Chromatography (HPLC). Analyses were performed with a LC10 HPLC UV system (Shimadzu, Tokyo, Japan), linked to a Class LC10 software (v1.4, Shimadzu, Tokyo, Japan, 1994) for data analysis. The column was a Teknokroma Kromosil 100 Si 25 cm × 0.46 nm, the UV wavelength was set at 302 nm, the mobile phase was dichlorometane/methanol 95/5 at 1 mL/min. ^1^H NMR spectra were recorded on a Jeol ECZ-R 600 (Jeol Ltd, Tokyo, Japan), at 600 and 150 MHz, respectively, using SiMe4 as the internal standard. The following abbreviations are used to designate peak multiplicity: s = singlet, d = doublet, t = triplet, m = multiplet, bs = broad singlet. ESI–MS spectra were recorded on a Micromass Quattro API micro (Waters Corporation, Milford, MA, USA) mass spectrometer. Data were processed using a MassLynx System (v4.1, Waters, 2009).

#### 2.2.2. Lipophilic Linker *N*-Octadecil-3-Maleimido-Benzamide (ST-MBS) Synthesis

ST-MBS was synthesized according to a method in the literature, slightly modified [14]. Amounts of 4.5 mg stearylamine and 5 mg MBS were dissolved in 1 mL CHCl_3_ and 15 μL di-triethylamine and were kept reacting at 40 °C for 4 h. The chloroform phase was extracted twice with 1% NaCl and twice with distilled water to remove water-soluble by-products. MgSO_4_ was added to the chloroform phase and filtered. The mixture was dried from chloroform using a rotor-evaporator. 

The reaction was assessed through Thin Layer Chromatography (TLC—acetic acid/chloroform/methanol 1/89/10). The product was characterized through HPLC. Analyses were performed with a YL9100 HPLC system equipped with a YL9110 quaternary pump, a YL9101 vacuum degasser and a YL9160 PDA detector, linked to YL-Clarity software for data analysis (v3.0.4.444, Young Lin, Hogye-dong, Anyang, Korea, 2009). The column was a Teknocroma C18 mediterranea Sea 25 × 0.46 cm, the PDA wavelengths were set at 220–290 nm, the eluent flow was 1 mL/min, and the gradient was 0 min: 100% water, 20 min: 100% acetonitrile, 30 min: 100% acetonitrile, and 35 min: 100% water. ^1^H NMR spectra were recorded on a Jeol ECZ-R 600, at 600 and 150 MHz, respectively, using SiMe4 as the internal standard. The following abbreviations are used to designate peak multiplicity: s = singlet, d = doublet, t = triplet, m = multiplet, bs = broad singlet. ESI–MS spectra were recorded on a Micromass Quattro API micro (Waters Corporation, Milford, MA, USA) mass spectrometer. Data were processed using a MassLynx System (Waters).

#### 2.2.3. PEGylated Linker Stearyl-PEG-Maleimide (ST-PEG-MBS) Synthesis

An in house engineered synthesis was developed for the ST-PEG-MBS. In the first step, 10 mg stearic acid (140 mM), 1.25 mg NHS (43 mM) and 1 mg EDC (26 mM) were dissolved in 0.25 mL DMF anhydrous and were kept reacting overnight at 45 °C. The reaction mixture was diluted with 1 mL chloroform and washed with 4 mL 0.1 M HCl to eliminate water soluble compounds. The organic phase was treated with MgSO_4_ and dried under nitrogen steam and checked through TLC (0.5% triethylamine in chloroform): being in excess of stearic acid, it was a mixture of the reagent and of activated stearic acid (36%). In a second step, 6.1 mg first step reaction product (corresponding to 2.2 mg activated stearic acid—12 mM), 2 mg MBS (12 mM), 12 mg diamino-PEG (12 mM) and 12 µL triethylamine (237 mM) were dissolved in 0.5 mL chloroform and were kept reacting overnight at 45 °C. The reaction mixture was dried under nitrogen steam, then the residue was dissolved in 400 µL ethanol, and 1.6 mL 1 M HCl was added to precipitate un-reacted stearic acid from the first step reaction. The obtained solution underwent size exclusion through a Sephadex G10 column to eliminate compounds with *M*_w_ < 500. ST-PEG-MBS was purified from un-reacted diamino-PEG through Dowex 50WX8-200 resin. The purified solution was freeze dried. The obtained conjugate (6.4 mg) was checked through TLC (acetic acid/chloroform/methanol 1/84/15) and characterized by ^1^H-NMR (300 MHz ^1^H was recorded on a Bruker 300 Avance instrument (Bruker, Billerica, MA, USA) at 25 °C). The product was characterized through HPLC. Analyses were performed with a YL9100 HPLC system equipped with a YL9110 quaternary pump, a YL9101 vacuum degasser and a YL9160 PDA detector, linked to YL-Clarity software for data analysis (Young Lin, Hogye-dong, Anyang, Korea), and, alternatively, with a LC10 HPLC UV system (Shimadzu, Tokyo, Japan) equipped with a ELSD detector, linked to a Class LC10 software for data analysis. The column was a Teknocroma C18 mediterranea Sea 25 × 0.46 cm, the PDA wavelengths were set at 220–290 nm, the eluent flow was 1 mL/min, and the gradient was 0 min: 100% water, 20 min: 100% acetonitrile, 30 min: 100% acetonitrile, and 35 min: 100% water.

#### 2.2.4. Protein Thiolation

##### Thiolated TRF (TRF-SH)

The thiolation of TRF was performed according to the literature with modifications [15]. A diluted solution of TCEP (5.7 µg/mL) in 0.1 M phosphate buffer at pH = 7.4 was prepared. Then, 2-iminothiolane was dissolved in this solution at various concentrations (ranging from 3.5 μg/mL to 14.0 µg/mL). To dissolve 1 mg TRF, 1 mL of this solution was employed and was kept reacting for 1 hour. Three different 2-iminothiolane/TRF molar ratios were employed: 2:1, 4:1, and 8:1. The TRF-SH underwent purification through size exclusion (Biogel P-6, BioRad, Hercules, CA, USA) and characterization through electrophoresis.

##### Thiolated INS (INS-SH)

The INS thiolation procedure was modified from the literature [16]. The 2-iminothiolane was dissolved in the above mentioned diluted TCEP solution at a concentration of 12.5 µg/mL, together with EDTA at a concentration of 150 µg/mL. INS was dissolved in this solution at a concentration of 0.5 mg/mL and was kept reacting for 1 h. The molar ratio between the peptide and 2-iminothiolane was 1:1. The INS-SH was purified through selective precipitation at pH = 5.0 and characterized through electrophoresis.

#### 2.2.5. SLN Preparation

According to the coacervation method described in a previous paper, 1% stearic acid SLNs were prepared [17]. Briefly, sodium stearate was dispersed in an aqueous solution of the polymeric stabilizer (PVA9000), and the mixture was then heated under stirring (300 rpm) up to a temperature of 50 °C to obtain a clear solution. A 1 M lactic acid solution (coacervating solution) was then added dropwise. The obtained suspension was then cooled in a water bath under stirring at 300 rpm until a temperature of 15 °C was reached.

The 1% behenic acid SLNs were prepared as follows. Sodium behenate was dispersed in water with PVA9000 and the mixture was then heated under stirring (300 rpm). A concentration of 1 M sodium hydroxide was added to obtain a completely clear micellar solution. First, 5 M ammonium chloride and then 1 M hydrochloric acid were added drop-wise to the mixture until complete behenic acid precipitation [18]. The obtained suspension was then cooled under stirring at 300 rpm until a temperature of 15 °C was reached.

ddMTX was dissolved in a small amount of hot ethanol and added to the micellar solution prior to acidification.

#### 2.2.6. SLN Functionalization

Maleimide linkers were dissolved in small amount of ethanol and added to the micellar solution prior to acidification. Purified thiolated protein solution was added to the SLNs and kept stirring overnight. Afterwards, the suspension was centrifuged at 26,000 rpm for 30 min (Allegra^®^ 64R centrifuge, Beckmann Coulter, Paolo Alto, CA, USA). The precipitate was re-suspended in 1% PVA9000 solution and protein grafting was assessed by electrophoresis. The obtained suspension was reacted overnight with an excess of reduced glutathione in order to saturate unreacted maleimide groups grafted onto the surfaces of SLNs, and, then, underwent size exclusion chromatography (Sepharose CL-4B, Sigma Aldrich, Saint Louis, MO, USA) to completely eliminate unbound proteins. The obtained sample was subject to electrophoresis, to estimate the amount of protein effectively bound to the SLNs.

#### 2.2.7. SLN Coating with PVA120000

The SLNs obtained through the coacervation method were stabilized by PVA9000. A suitable procedure was set up to obtain PVA120000 coating. Briefly, the ddMTX-loaded SLNs, plain or functionalized with proteins, were centrifuged at 26,000 rpm for 30 min (Allegra^®^ 64R centrifuge, Beckmann Coulter, Paolo Alto, CA, USA). The precipitate was concentrated to 2.5% lipid through re-suspension in a 25 mg/mL PVA120000 solution.

#### 2.2.8. SLN Characterization

The particle size of the SLNs and polydispersity index were determined 1 h after preparation using the dynamic light scattering (DLS) technique (90Plus, Brookhaven Instrument Corporation, Holtsville, NY, USA). Size measurements were obtained at an angle of 90° at 25 °C. All data were determined in triplicate.

The ddMTX content in the suspension was determined through fluorescence RP-HPLC after a 500-fold water dilution and derivatization.

The amount of protein grafted onto the surfaces of the SLNs was determined through electrophoresis and densitometry.

Preliminary drug release experiments and SLN stability studies were performed in cell culture medium and plasma (Supplementary Materials, Figure S2, Table S1).

#### 2.2.9. Sodium Dodecyl Sulfate Polyacrylamide Gel Electrophoresis (SDS_PAGE)

To verify the effective functionalization of the SLNs, 10 µL TRF-SH and INS-SH SLN, mixed with 5 µL Laemly Buffer (60 mmol/L Tris-Cl, pH 6.8; 2% *w*/*v* SDS; 10% *v*/*v* glycerol, 5% *v*/*v* β-mercaptoethanol; 0.01% *w*/*v* bromophenol blue) were separated by SDS-PAGE. 10 µg purified TRF or INS, diluted in 10 µL Laemly Buffer, were loaded as the internal standard. The electrophoresis was performed without heating samples, to avoid TRF or INS denaturation and/or detachment from the SLNs, loading samples onto a 12% (for TRF-SLNs) or 15% SDS-polyacrylamide (for TRF-SLNs) running gel (Bio-Rad). The running conditions were 100 V for 1 h. After the electrophoretic separation, to detect the free TFR or INS, or the protein grafted onto SLNs, the gels were stained with 10 mL Coomassie BrilliantBlue solution, that can detect 100–500 ng of protein per spot [19] (0.2% *w*/*v* Coomassie Blue, 7.5% *v*/*v* acetic acid and 50% *v*/*v* ethanol), for 1 h at room temperature, followed by overnight de-staining with deionized water. Densitometry was performed with ImageJ 1.50i Software (National Institute of Health, Bethesda, MD, USA, 2016).

#### 2.2.10. Permeability through hCMEC/D3 Cell Monolayer

hCMEC/D3 cells, seeded as reported above in Transwell devices, were incubated at day 7 with either the free drug (MTX) or the SLN-entrapped drug (ddMTX-loaded SLNs), in the experimental conditions described in the Results section. The medium in the lower chamber was then collected and the amount of the drug was measured through fluorescence RP-HPLC.

#### 2.2.11. In Vivo Biodistribution

The ddMTX-loaded SLNs, and disodium MTX (1 mg/kg) solution in normal saline, were administered through a catheter surgically positioned in the jugular vein of male Wistar healthy rats (weight, 250 g) [20]. At scheduled times, the rats were sacrificed by CO_2_-induced euthanasia; plasma was withdrawn, and the organs (liver, spleen, kidneys, lungs, heart and brain) were removed surgically. Blood samples were collected in heparinized tubes and centrifuged to isolate plasma. Urine was withdrawn through a syringe from the bladder. The brain underwent capillary depletion to isolate brain capillaries from parenchyma. Briefly, freshly removed brains were manually homogenized with a potter in 3.5 mL Phosphate Buffered Saline (PBS) buffer; then 1 mL of the homogenate was diluted with 1.16 mL dextran 60,000–90,000 and underwent gradient centrifugation at 4000× *g* for 10 min at 25 °C in a test tube (Allegra^®^ 64R centrifuge, Beckmann Coulter, Paolo Alto, CA, USA). The capillaries were isolated from the bottom of the tube [21]. Other organs were homogenized with UltraTurrax® (IKA, Staufen, Germany) for 5 min in water at a tissue concentration of 125 mg/mL; tissue homogenates and plasma underwent the derivatization reaction prior to fluorometric HPLC detection described below.

Each experiment was performed with four rats for each experimental condition (sample size calculated with G*Power 3.1.9.2, Universitat Kiel, Kiel, Germany, 1992).

#### 2.2.12. Enzymatic Synthesis of 7-Hydroxymethotrexate (7OH-MTX)

##### Isolation of Aldehyde Oxidase from Rabbit Liver

The rabbits were sacrificed in a slaughterhouse for food purposes. The liver was isolated and kept in an ice bath for 10 min prior to manipulation in the laboratory. Aldehyde oxidase was isolated from liver according to a literature method with slight modifications [22,23]. An amount of 10 g of tissue was homogenized with UltraTurrax (Ika, Staufen, Germany) in 1.15% KCl in an ice bath. The homogenate was centrifuged at 10,000× *g* for 30 min at 4 °C (J2-21 centrifuge, Beckmann Coulter, Paolo Alto, CA, USA). The supernatant was separated and heated to 55 °C for 10 min: after rapid cooling, a precipitate was obtained and it was isolated through centrifugation at 15,000× *g* for 45 min at 4 °C (J2-21 centrifuge, Beckmann Coulter, Paolo Alto, CA, USA). The supernatant was isolated, ammonium sulphate was added in order to give 50% saturation, and the mixture was gently shaken at 4 °C for 20 min and successively centrifuged at 6000× *g* for 30 min at 4 °C (J2-21 centrifuge, Beckmann Coulter, Paolo Alto, CA, USA). The precipitate was suspended in 5 mL 0.05 M buffer phosphate pH = 7.8 with 0.1 mM EDTA. The same buffer was employed for elution on a Sephadex G75 column. The fractions showing significant absorbance at λ = 280 nm were put together and successively fractioned in aliquots, and kept at −80 °C until usage. Protein determination in the enzyme solution was performed using the Folin–Ciocolteu method, employing bovine albumin as the standard. 

The isolated enzyme corresponds to a mixture of aldehyde oxidase and xanthine oxidase: the first is the major component involved in MTX oxidation, but, given that the second could contribute, no further purification was performed.

The activity of the enzyme was determined spectrophotometrically (Cary 50 Bio, Varian, Paolo Alto, CA, USA) at 37 °C in 67 mM phosphate buffer at pH = 7.0. The oxidation rate of 3-methylisoquinoline (0.250 mM) was monitored at 420 nm by following potassium ferricynide reduction [24].

##### 7OH-MTX Synthesis from MTX

Synthesis was performed according to the literature with modifications [25]. Briefly, 5 mg MTX were dissolved in 33 mL 0.133 M Sorensen buffer brought to pH = 7.8 with 5 M sodium hydroxide. An amount of 10 mL enzyme solution (corresponding to 112 mg enzyme) was slowly unfrozen in an ice bath and then added to the MTX solution. The mixture was kept at 37 °C for 2 h. Afterwards the pH was brought to 8.4 with 0.5 M sodium hydroxide and kept at 40 °C overnight. The mixture was centrifuged at 5000 rpm (Rotofix 32A centrifuge, Hettich, Tuttlingen, Germany) and the supernatant was isolated, heated until 70 °C and centrifuged. Complete deproteinization was obtained with Amicon Centriflo (Millipore, Burlington, MS, USA) equipped with 50,000 Da dialysis membrane. The dialyzed solution was brought to pH = 4.0 with acetic acid and kept at 4 °C overnight in order to obtain the complete precipitation of 7OH-MTX. The obtained suspension was centrifuged at 25,000 rpm (Allegra^®^ 64R centrifuge, Beckmann Coulter, Paolo Alto, CA, USA), the supernatant was discarded and the precipitate was dried under vacuum. Nearly 2 mg 7OH-MTX was obtained. The reaction product was assessed through PDA–HPLC described below and the eluted fractions were collected and analyzed with mass spectroscopy (Micromass Quattro microTM API, Waters Corporation, Milford, MA, USA).

#### 2.2.13. MTX, 7OH-MTX and ddMTX HPLC Analysis

Analyses were performed with a YL9100 HPLC system equipped with a YL9110 quaternary pump, a YL9101 vacuum degasser and a YL9160 PDA detector and/or a Shimadzu RF-10A fluorescence detector (Shimadzu, Tokyo, Japan), linked to YL-Clarity software for data analysis (Young Lin, Hogye-dong, Anyang, Korea).

##### (Photo Diode Array Detector) PDA HPLC of MTX and 7OH-MTX

HPLC analysis was performed with Mediterranea Sea RP 2.5 μm 250 × 0.46 cm column (Tecnokroma, Barcelona, Spain). The mobile phase was ammonium acetate 0.1 M/methanol/acetonitrile (87/5/8), used at 1 mL/min flow. The PDA detector was set at λ = 305 nm. The retention times were 17.0 and 20.0 min for MTX and 7OH-MTX, respectively.

##### Derivatization and Fluorescence HPLC of MTX and ddMTX

The RP-HPLC method was developed for the determination of both MTX and ddMTX in biological samples, based on a method in the literature [26]. Briefly, 0.25 mL of samples was added to 0.1 mL 10% SDS aqueous solution, in order to promote ddMTX dissolution. Then, they were deproteinized with 0.08 mL of 10% trichloroacetic acid (TCA) and centrifuged. An amount of 0.2 mL of the supernatant was added to 0.05 mL 5 M pH 5.0 acetate buffer. MTX or ddMTX were then oxidized to fluorescent pterydin carboxylic acid with 0.05 mL 5% KMnO_4_. HPLC analysis was performed with Mediterranea Sea RP 2.5 μm 150 × 4.6 mm column (Tecnokroma, Barcelona, Spain). Here, 0.05 M pH 6.7 TRIS buffer (adjusted with phosphoric acid) was used as a mobile phase at 1 mL/min flow. After peak elution, the gradient was performed with 30% acetonitrile in order to clean the column from the reaction sub-products. Folic acid was employed as the internal standard. The retention time was 12 min for MTX. *R*^2^ 0.99, Limit of Detection (LOD) 0.01 µM, Limit of quantification (LOQ) 0.03 µM, Bias 3%, Coefficient of Variation (CV) 5.3%, Recovery 87%.

##### Ultra-High Performance Liquid Chromatography (UPLC–MS) Analysis of MTX and its Metabolites

The derivatized samples also underwent UPLC–MS analysis according to the following conditions. UPLC–MS analysis was performed with an Acquity UPLC (Waters Corporation, Milford MA, USA), equipped with Binary Solvent Manager (BSM), Sample Manager (SM), Column Manager (CM) and PDA detectors. The analytical column was a Zorbax Eclipse XDB-C18 150 × 4.6 mm. The mobile phase consisted of methanol and ammonium acetate buffer pH = 6.0 (30/70). UPLC retention time (R_t_) was obtained at flow of 0.4 mL/min, and the column effluent was monitored using Micromass Quattro micro^TM^ API Esci multi-mode ionization enabled as detector. The MS conditions were: purging gas (nitrogen) heated at 350 °C, at a flow rate of 800 L/h; nebulizer gas (nitrogen) at 80 L/h; capillary voltage in the negative mode at 3000 V; fragmentor voltage at 20 V.

#### 2.2.14. Statistical Analysis

Results were reported as mean ± standard deviation, and statistical analysis was performed using a two-tailed Student’s t-test (Prism Graphpad 7.0, Graphpad Software, San Diego, CA, USA, 2016).

## 3. Results

The ddMTX-loaded stearic acid and behenic acid SLNs were formulated according to the fatty acid coacervation technique [17]. The ddMTX characterization is shown in the Supplementary Materials. The ddMTX-loaded SLN composition and characterization are shown in Table 1. The behenic acid SLNs were prepared according to an optimized composition, in order to obtain a more homogeneous nanosuspension with a reduced polydispersity and particle size [18]. PVA120000 was also employed as a suspending/coating agent, as an alternative to PVA9000, in order to improve biodistribution in the plasma compartment: in this case, PVA9000 was removed by centrifugation and the SLNs were re-suspended in a PVA 120000 solution. Re-suspension in PVA120000 resulted in a slight increase in particle size, probably due to the viscosity of the polymer.

**Figure 1:**
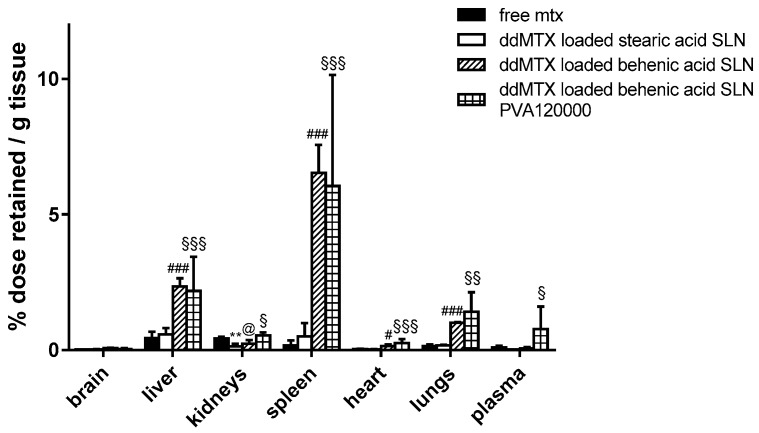
Biodistribution of the unfunctionalized ddMTX-loaded SLNs and free MTX (sacrifice 30 min after administration). Statistical analysis: ddMTX-loaded SLNs vs. free MTX; stearic acid SLNs < MTX: *p* < 0.005 **; behenic acid SLNs < MTX: *p* < 0.05 @; behenic acid SLNs > MTX: *p* < 0.05 #, *p* < 0.001 ###; behenic acid SLNs PVA120000 > MTX: *p* < 0.1 §, *p* < 0.01 §§, *p* < 0.05 §§§.

Preliminary biodistribution experiments were performed by sacrificing the animals 30 min after the IV administration of ddMTX-loaded SLNs, aiming to compare drug accumulation in the tissues between SLNs with different lipid matrixes/stabilizing polymers and free MTX. A higher persistence in tissues was noted when the drug was loaded in behenic acid SLNs compared to free MTX, except for the kidneys. However, a higher plasmatic concentration is necessary in order to optimize the contact with BBB cells and favor its overcoming. The employment of PVA120000 as a suspending agent led to an increased drug concentration in plasma (Figure 1). Thus, the ddMTX-loaded behenic acid SLNs stabilized with PVA120000 were employed for the following studies with functionalized SLNs.

Functionalization with proteins was achieved by grafting a maleimide moiety on the nanoparticle’s surface through a properly synthesized linker, and exploiting its reactivity towards the thiolated proteins. The derivatization was confirmed by SDS-PAGE followed by Blue Coomassie staining. The ST-MBS and ST-PEG-MBS characterizations are shown in the Supplementary Materials (Figure S1).

**Figure 2:**
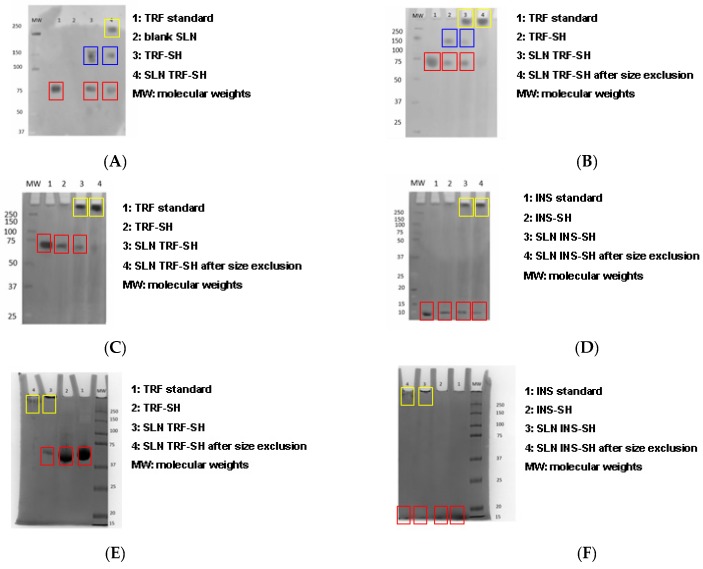
Electrophoresis. Red boxes: free proteins; blue boxes: dimerized proteins; yellow boxes: proteins grafted onto SLNs. (**A**) ST-MBS; 8:1 2-iminothiolane/TRF molar ratio; (**B**) ST-MBS; 4:1 2-iminothiolane/TRF molar ratio; (**C**) ST-MBS; 2:1 2-iminothiolane/TRF molar ratio; (**D**) ST-MBS; 1:1 2-iminothiolane/INS molar ratio; (**E**) ST-PEG-MBS; 2:1 2-iminothiolane/TRF molar ratio; (**F**) ST-PEG-MBS; 1:1 2-iminothiolane/INS molar ratio.

The preliminary studies were performed in order to optimize the thiolation of proteins. Electrophoresis was carried out to investigate the effect of 2-iminothiolane/TRF molar ratio on the protein aggregation caused by disulfide bond formation (Figure 2A lane 3, Figure 2B,C,E, lanes 2). Using the 8:1 and 4:1 2-iminothiolane/TRF molar ratios, TRF-SH was present in its dimerized form (Figure 2A,B blue boxes; 150 kDa band): this indicates protein aggregation due to disulfide bond formation. Disulfide bonds reduce the efficacy of following protein grafting onto SLNs through reaction with maleimide linkers. Thus, a 2:1 molar ratio was employed, allowing plain TRF-SH retention (Figure 2C,E lanes 2 red boxes; 75 kDa band). Since the dimerization of INS-SH was less detectable by electrophoresis (Figure 2D,F), a 1:1 2-iminothiolane/INS molar ratio was used, in order to minimize the possibility of disulfide bond formation.

The composition and characterization of ddMTX-loaded behenic acid SLNs, functionalized with proteins, is shown in Table 2.

In electrophoresis, proteins grafting onto the surfaces of SLNs caused protein retention at the top of the running gel [9], because of the larger size of the nanoparticles compared to proteins (Figure 2A lane 4, Figure 2B–F lanes 3 yellow boxes). Blank SLNs were not detected in electrophoresis, as expected (Figure 2A lane 2). Both ST-MBS and ST-PEG-MBS were allowed to graft proteins onto the surfaces of SLNs, even if a higher amount was grafted with ST-MBS. Moreover, after size exclusion, the proteins remained grafted onto the surfaces of SLNs almost completely when ST-MBS was used (Figure 2C,D lanes 4 yellow boxes), while with ST-PEG-MBS a certain protein leakage was noted (Figure 2E,F lanes 4 yellow boxes), probably due to the major hydrophilicity of ST-PEG-MBS. Insulin grafting was more labile and a small amount of free protein was also recovered in samples purified by size exclusion (Figure 2D,F lanes 4 red boxes). Since a significantly lower amount of protein was grafted onto SLNs with ST-PEG-MBS compared to ST-MBS, in the definitive preparation procedure a lower amount of thiolated protein was employed for reaction with the first one, in order to avoid waste of reagents (Table 2).

**Figure 3:**
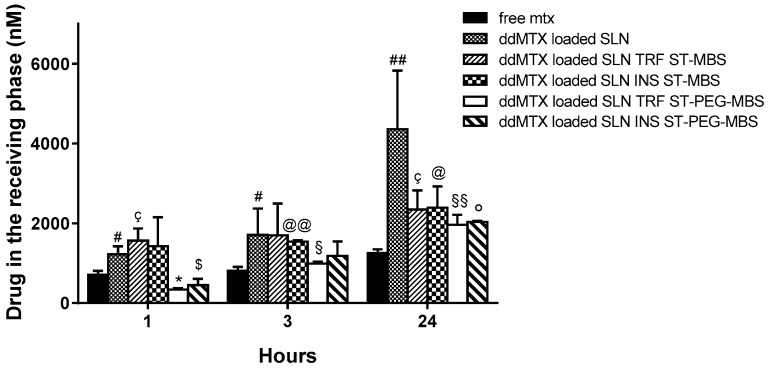
Permeation through the hCMEC/D3 cells monolayer of unfunctionalized and functionalized ddMTX-loaded behenic acid SLNs and free MTX. Statistical analysis: ddMTX-loaded SLNs vs. free MTX; SLNs TRF ST-PEG-MBS<MTX: *p* < 0.05 *; SLNs INS ST-PEG-MBS<MTX: *p* < 0.1 $; SLNs > MTX: *p* < 0.1 #, *p* < 0.05 ##; SLNs TRF ST-PEG-MBS>MTX: *p* < 0.1 §, *p* < 0.05 §§; SLNs INS ST-PEG-MBS > MTX: 24 h *p* < 0.005 °; SLNs TRF ST-MBS>MTX: *p* < 0.05 ç; SLNs INS ST-MBS > MTX: *p* < 0.05 @, *p* < 0.005 @@.

In vitro permeation studies on cellular models of BBB were performed in order to investigate the ability of SLNs to target BBB endothelial cells (Figure 3). Unfunctionalized SLNs showed a significantly increased delivery across the hCMEC/D3 cells monolayer, compared to free MTX. SLNs functionalized through the ST-MBS linker were delivered more than the free MTX at all time points. SLNs functionalized through the ST-PEG-MBS linker showed a lower delivery at a short incubation time (1 h); after 24 h incubation, however, the delivery was higher than the free drug. Despite being an unexpected result, the lower permeation of functionalized SLNs compared to unfunctionalized SLNs at 24 h might be explained through the saturation of a receptor-mediated endocytosis mechanism.

**Figure 4:**
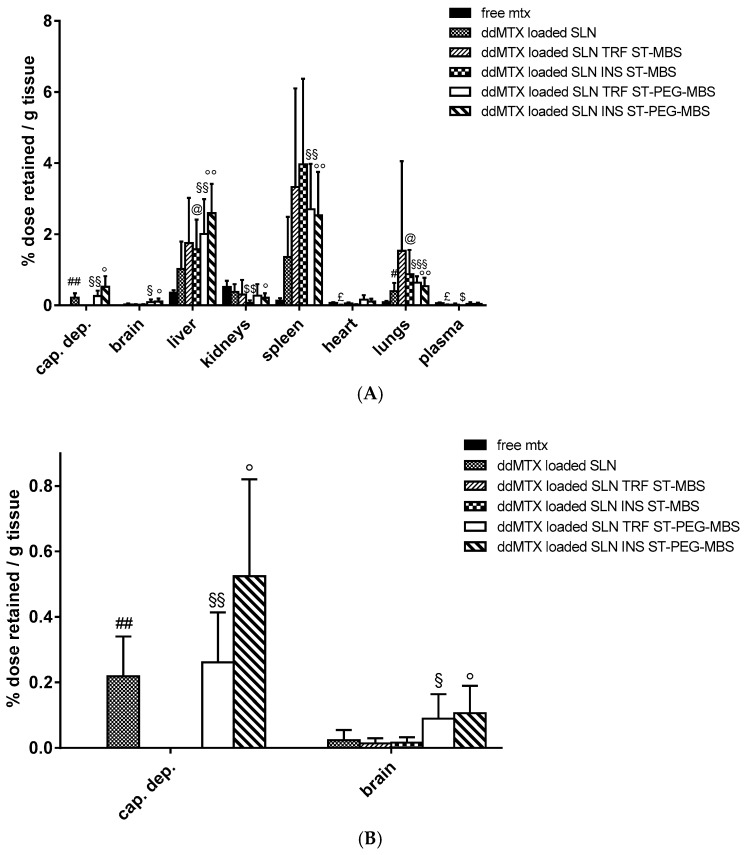
Biodistribution of functionalized ddMTX-loaded SLNs and free MTX (sacrifice 3 h after administration): in (**A**) the whole body and (**B**) brain and capillary depletion (cap. dep.). Statistical analysis: ddMTX-loaded SLNs vs. free MTX; SLNs < MTX: *p* < 0.01 £ SLNs INS ST-PEG-MBS < MTX: *p* < 0.05 *; SLNs INS ST-MBS < MTX: *p* < 0.01 $, p < 0.05 $$; SLNs > MTX: *p* < 0.1 #, *p* < 0.05 ##; SLNs TRF ST-PEG-MBS > MTX: *p* < 0.1 §, *p* < 0.05 §§, *p* < 0.01 §§§; SLNs INS ST-PEG-MBS>MTX: *p* < 0.1 °, *p* < 0.05 °°; SLNs TRF ST-MBS > MTX: *p* < 0.1, ST-MBS > MTX: *p* < 0.1 @.

In vivo biodistribution experiments were performed with functionalized ddMTX-loaded SLNs in order to further investigate the role of protein functionalization on brain accumulation. In this case, animal sacrifice was done 3 h after IV administration in order to allow the prolonged contact of nanoparticles with BBB cells, thus optimizing brain accumulation. Moreover, the capillary depletion method was used to isolate brain endothelial cells. Obtained data were compared to free MTX (Figure 4). SLN functionalization with proteins increased drug accumulation in lungs, liver and spleen (Reticulo Endothelial System—RES organs), regardless of the linker employed. However, accumulation in brain parenchyma was significantly lower compared to non-target organs; a significant increase of brain accumulation was noted for SLNs functionalized with ST-PEG-MBS. Accumulation in brain capillaries was promising for unfunctionalized SLNs and SLNs functionalized with ST-PEG-MBS, while almost absent when ST-MBS was employed (Figure 4B). 

Finally, after the administration of ddMTX-loaded SLNs, the presence of MTX and 7OH-MTX was qualitatively investigated, because they can contribute to the pharmacological activity. Preliminary evidence was obtained from UPLC–MS: analyses were performed on urine of treated animals (shown in the Supplementary Materials), that is the main site of accumulation of drug metabolites. MTX and 7OH-MTX were identified as ddMTX metabolites (see Supplementary Materials), confirming the prodrug hydrolysis and the parent drug metabolism [27,28]. Among the metabolites of MTX, 7OH-MTX is the most known active one [27,28]. Thus, a suitable enzyme-assisted procedure [25] was employed to synthesize the metabolite starting directly from MTX: this method is solvent free and allows avoiding the multi-step process required by chemical synthesis [29]. The results are shown in the Supplementary Materials. The synthesized drug allowed to confirm the identification of the metabolite in UPLC–MS.

## 4. Discussion

The SLNs obtained using the fatty-acid coacervation method were loaded with ddMTX, an ester prodrug of MTX that is active towards GB in vitro [8]. A range of factors that influence brain biodistribution in vivo were considered.

Two lipid matrixes (stearic acid and behenic acid) were compared. An optimized formulation technique for behenic acid SLNs was employed [18] in order to improve the homogeneity of the nanoparticle suspensions.

Preliminary biodistribution experiments, performed via animal sacrifice 30 min after administration, showed that ddMTX-loaded behenic acid SLNs have higher drug persistence in tissues than free MTX (Figure 1). Nevertheless, with ddMTX-loaded behenic acid SLNs, the drug level in plasma was low. Reduced particle size is an important parameter that contributes to prolonging nanoparticle circulation in the bloodstream. In fact, nanoparticles with a diameter greater than 200 nm activate the complement system and are quickly removed from the bloodstream, accumulating in the liver and spleen more rapidly compared to smaller ones [30]. There is still debate as to whether the rapid accumulation is due to simple filtration or increased binding opportunities between the mononuclear cells and the nanoparticles [31]. However, despite being solvent free, easy to scale-up and versatile [17], the coacervation technique struggles to provide SLNs with a mean diameter below 200 nm. On the other hand, surface lipophilicity increases the nanoparticle removal rate from the bloodstream, thus they are frequently coated with hydrophilic polymers, such as polyethylene glycol (PEG) to avoid the RES system and increase blood circulation half-life, despite the fact that coating with PEG reduces the surface charge and negatively affects the cellular uptake [32]. Therefore, we focused on improving the hydrophilic shell surrounding the SLNs, which should hamper opsonization. Apart from PEG, other polymers forming a hydrophilic shell around the particles can be employed, such as PVA, showing an improved efficacy by increasing molecular weight [33]. The SLNs produced by coacervation were coated with PVA9000, a hydrophilic polymer, whose reduced molecular weight, however, favors its rapid elimination by the kidneys [34]. Re-suspending the SLNs in PVA120000 led to higher drug levels in the plasma, despite a slight increase in the mean diameter of the nanoparticles (Figure 1). Its high molecular weight means that PVA120000 is eliminated by the kidneys more slowly than PVA9000. This fact might stabilize the nanoparticles in the bloodstream and it is in agreement with the literature data [33]. Being coated and sterically stabilized by the non-ionic PVA, the SLNs by coacervation did not show any Zeta potential (data not shown). Nevertheless, their mean size increased when resuspended in PVA120000, probably because of its higher molecular weight. Thus, even if the particle size increased, the concentration in the bloodstream increased, probably because of the effect of PVA120000 on opsonization. 

The ddMTX-loaded behenic acid SLNs that were stabilized with PVA120000 were functionalized with two proteins (TRF or INS), whose receptors are abundantly expressed in BBB endothelial cells. The proteins were thiolated and grafted, through either a lipophilic (ST-MBS) or a PEGylated (ST-PEG-MBS) linker, both of which expose a maleimide moiety on the surfaces of SLNs. ST-MBS was synthesized according to a method in the literature [14]. ST-PEG-MBS was synthesized and characterized in house (Supplementary Materials). According to ^1^H NMR, the maleimide protons were detected as a multiplet at 6.5 ppm and the stearic moiety could be easily evidenced at high field (1.2 ppm multiplet for the alkylic chain and 0.9 ppm for the methyl moiety). Both maleimide and stearic moiety were linked to diamino-PEG in a 1:1 molar ratio. The thiolation of proteins is frequently associated with aggregation due to the formation of disulfide bonds: to this aim, EDTA is frequently used to scavenge metals that favor thiol oxidation. EDTA, however, cannot be employed with TRF because it would sequestrate iron, leading to so-called holo-TRF, which possesses a low affinity for the TRF receptor. A mild reducing agent, TCEP, was therefore used to inhibit thiol oxidation. This decision was made on the basis of the literature data [15]. The 2-iminothiolane/protein ratio was also optimized in order to avoid protein aggregation (Figure 2). The functionalization and purification of the SLNs were followed step-by-step through electrophoresis. Size exclusion was employed to eliminate free proteins. The unreacted maleimide moiety present on the surfaces of SLNs was saturated with an excess of reduced glutathione in order to avoid the targeting of the heart and muscle (data not shown) that would occur because of a reaction between maleimide and the free thiol group of myosin [35].

The first evaluation of functionalized SLNs was performed in vitro (Figure 3). Indeed, ddMTX has a poor permeability across BBB [8] due to the presence of ATP binding cassette transporters on the luminal side such as P-glycoprotein (Pgp), Multidrug resistance Related Proteins (MRP) 1, 2 and 4 and the Breast Cancer Resistance Protein (BCRP) that extrude the MTX derivatives from endothelial cells back to the blood stream [36]. By contrast, we recently demonstrated that the loading of ddMTX onto SLNs significantly increased drug delivery across the BBB [8]. If not functionalized, SLNs—as most nanoparticles and/or liposomes—are subjected to macropynocytosis that allows an efficient route of internalization of SLNs. The internalization is followed by the fusion of macropynocytic vesicles with endosomal/lysosomal compartments and the release of SLN cargo within the cytosol [37]. After this step, the released ddMTX may be effluxed towards brain parenchyma by MRP3, an ABC transporter located on the basolateral membrane of BBB that transports MTX-derivatives [36]. The sum of the increased endocytosis, subtraction of ddMTX from the backward efflux through Pgp, MRP1, MRP2, MRP4 and BCRP, and the increased basolateral efflux of ddMTX through MRP3 following the endocytosis of ddMTX-SLNs may justify the increased delivery of the compound when loaded onto SLNs compared to delivery obtained as free drug. Paradoxically, protein grafting onto SLNs makes SLN permeation slower. This can be due to the fact that the functionalized SLNs exploit a receptor-mediated system, which undergoes saturation. Moreover, one of the mechanisms of SLN internalization can be the endocytosis mediated by clathrin-coated vesicles. This mechanism has been demonstrated for instance for the transcellular uptake of apoE-conjugated SLNs in BBB cells, where it represents the predominant transcellular pathway of internalization [38]. We cannot exclude that the lower delivery of conjugated SLNs versus unconjugated SLNs was also due to the saturation of clathrin-dependent endocytosis in our system. Whatever the mechanism is, this effect is more evident in SLNs that are functionalized with ST-PEG-MBS, but also occurs when ST-MBS is employed. It is important to note, however, that all the SLN formulations provided higher drug delivery across the BBB after 24 h than the free drug. 

Frequently, the biodistribution of nanoparticles showed a strong correlation to nanoparticle size with the smaller sized particles that are able to pass the BBB [32]. However, the capability of polybutylcyanoacrylate nanoparticles larger than 200 nm to successfully overcome BBB is documented by a recent publication [39]. Interestingly, the nanoparticles’ surface chemical properties, and not particle size, resulted to be the parameter with the most significant influence on BBB passage. Different factors at the target site (the BBB) and in the bloodstream should be taken in account. In vitro experiments with BBB cell models mimic the target site and demonstrate that all the SLN formulations under study, functionalized or not, provided drug delivery across the BBB according to endocytosis mechanisms, regardless of their size. The in vivo fate of nanoparticles is more complex and depends on the interaction with bloodstream components. Having a high concentration of nanoparticles in the blood stream means that the nanoparticles have more contact with the BBB endothelial cells, favoring the endocytosis process [40]. This is a relevant factor that, in the case of SLNs stabilized with PVA120000, can influence the ability to overcome the BBB. However, we are aware that in vitro experiments on cellular BBB models are not fully comparable with in vivo experiments. In fact, drug distribution and elimination phases are not present in the Transwell system and the formulations are kept in contact with the endothelial cell monolayer for a longer time than in in vivo conditions, enhancing non-specific endocytosis phenomena. In vivo, instead, rapid clearance of SLNs from the blood stream avoids receptor saturation, emphasizing the role of specific SLN functionalization.

We then moved to the more physiological in vivo setting. Preliminary experiments with unfunctionalized SLNs stabilized with PVA120000 showed promising drug concentrations in plasma 30 min after administration (Figure 1). Consequently, we hypothesized that a longer time frame after administration would have prolonged the contact between the SLNs and the BBB, thus optimizing endocytosis by the endothelial cells. Thus, biodistribution experiments on functionalized ddMTX-loaded SLNs were performed by sacrificing the animals 3 h after administration of the formulations under study. The drug concentration in the brain was lower than that in non-target tissues. However, interesting differences between ST-MBS and ST-PEG-MBS grafted SLNs were noted (Figure 4). Although a higher protein amount was grafted with ST-MBS (Table 1 and Table 2), brain biodistribution was improved only when ST-PEG-MBS was used. Furthermore, capillary depletion showed no endothelial-cell internalization for ST-MBS-grafted SLNs and good uptake for ST-PEG-MBS-grafted SLNs. The presence of a PEG spacer between the nanoparticles and the targeting protein is considered important for the interaction with the receptor [41]. Moreover, TRF and INS receptors are expressed not only in the BBB endothelial cells, but also in various tissues (including macrophages and RES organs), while the protein grafting of a nanoparticulate system is thought to increase its immunogenicity. Unpredictable variations in in vivo biodistribution should thus be expected. It has been reported that the protein density on the nanoparticle’s surface and the strength of the interaction between the ligand and the receptor in the target tissue play a key role for protein-targeted nanoparticles [42]; selective targeting to endothelial cells is obtained when a lower amount of protein is grafted onto the nanoparticles, while a higher amount generally drives towards macrophages and RES [43]. In fact, ST-PEG-MBS grafting should target SLNs to the BBB, most likely because of the improved interactions with the receptor and the reduced amount of protein grafted onto the surfaces of SLNs. ST-MBS grafting, instead, probably addresses mainly towards non-target organs. This difference was not observed to be so strong in in vitro experiments, where only the receptors that are expressed on endothelial cells are involved, and interactions with non-target organs, which occur in vivo, are excluded.

The ddMTX prodrug was metabolized in vivo: MTX and 7OH-MTX were identified in animals administered with ddMTX-loaded SLNs. Indeed, the metabolism plays a key role in the clearance of the drug. With the liver being the main organ of accumulation, it can be the subject of off-target toxic effects. Thus, future studies will also need to evaluate the biodistribution of the drug and its metabolites at longer time points after administration (i.e., 24 h), in order to investigate the safety of the formulation.

## 5. Conclusions

In this experimental work, ddMTX-loaded SLNs were formulated, surface engineered and functionalized with TRF and INS, and then tested in vitro and in vivo to overcome the BBB. Functionalization was achieved by grafting onto the nanoparticle’s surface a maleimide linker and exploiting its reactivity towards thiolated proteins. The reaction conditions were optimized in order to avoid protein aggregation due to disulfide bond formation. The employment of ST-PEG-MBS transported SLNs towards the BBB in vivo, while ST-MBS transported SLNs towards non-target organs, probably because of the different amounts of protein bound onto the surfaces of SLNs. The in vitro data on cells might suggest that such formulations overcome the BBB according to receptor-mediated endocytosis that can undergo saturation. On the basis of the promising results in terms of biodistribution, we are going to perform further studies on in vivo glioma models in order to evaluate the potential application of functionalized SLNs to GB therapy.

## Figures and Tables

**Table 1:** Composition and characterization of the unfunctionalized ddMTX-loaded SLNs.

Lipid Matrix	Stearic Acid	Behenic Acid	
Stabilizer	PVA9000	PVA9000	PVA120000
**Composition**			
Sodium stearate (mg)	25	-	-
Sodium behenate (mg)	-	25	25
PVA9000 (mg)	25	50	50 ^1^
PVA120000 (mg)	-	-	25
1 M Lactic acid (µL)	125	-	-
1 M sodium hydroxide (µL)	-	30	30
5 M ammonium chloride (µL)	-	65	65
1 M hydrochloric acid (µL)	-	100	100
ddMTX (mg)	2.0	2.0	2.0
Distilled water (mL)	2.5	2.5	1 ^2^
**Characterization**			
Mean size (nm)	353 ± 35	351 ± 45	435 ± 33
Polydispersity	0.240	0.155	0.250
ddMTX (mg/mL)	0.71	0.78	0.81

^1^ partially removed after centrifugation; ^2^ after centrifugation and resuspension in PVA120000 solution

**Table 2:** Composition and characterization of the functionalized ddMTX-loaded behenic acid SLNs.

Linker	ST-MBS		ST-PEG-MBS	
Protein linked	TRF	INS	TRF	INS
**Composition**				
Sodium behenate (mg)	25	25	25	25
PVA9000 (mg)	50 ^1^	50 ^1^	50 ^1^	50 ^1^
PVA120000 (mg)	25	25	25	25
1 M sodium hydroxide (µL)	30	30	30	30
5 M ammonium chloride (µL)	65	65	65	65
1 M hydrochloric acid (µL)	100	100	100	100
ddMTX (mg)	2.0	2.0	2.0	2.0
ST-MBS (mg)	0.2	0.2	-	-
ST-PEG-MBS (mg)	-	-	1.0	1.0
TRF-SH (mg)	3.6	-	1.0	-
INS-SH (mg)	-	3.6	-	1.0
Distilled water (mL)	1 ^2^	1 ^2^	1 ^2^	1 ^2^
**Characterization**				
Mean size (nm)	437 ± 55	429 ± 8	500 ± 45	445 ± 41
Polydispersity	0.122	0.222	0.209	0.103
ddMTX (mg/mL)	0.95	0.36	0.51	0.41
Protein linked (µg/mg lipid)	27.8	18	3	2.2

^1^ partially removed after centrifugation, ^2^ after concentration through centrifugation and resuspension

## Supplementary Materials

The following are available online at http://www.mdpi.com/1999-4923/11/2/65/s1, Figure S1: ^1^H-NMR spectrum of ST-PEG-MBS, Figure S2: Drug release from ddMTX loaded behenic acid SLN, Table S1: Particle size of ddMTX loaded behenic acid SLN in cell culture medium and rat plasma.
