# Supplementary material for: Methotrexate-Loaded Solid Lipid Nanoparticles: Protein Functionalization to Improve Brain Biodistribution
Source: Pharmaceutics. 2019 Feb 2;11(2):65. doi: 10.3390/pharmaceutics11020065 (PMC6409770; doi:10.3390/pharmaceutics11020065)
Supplement: Supplementary file 1 [file pharmaceutics-11-00065-s001.pdf]

Supplementary Materials: Methotrexate Loaded Solid Lipid Nanoparticles: Protein Functionalization to Improve Brain Biodistribution

Elisabetta Muntoni, Katia Martina, Elisabetta Marini, Marta Giorgis, Loretta Lazzarato, Iris Chiara Salaroglio, Chiara Riganti, Michele Lanotte and Luigi Battaglia

Didoceylmethotrexate (ddMTX) Synthesis and Characterization S1

Lipophilic Linker *N*-Octadecil-3-Maleimido-Benzamide (ST-MBS) Synthesis and Characterization S1

PEGylated Linker Stearyl-PEG-Maleimide (ST-PEG-MBS) Synthesis and Characterization S2

7-Hydroxymethotrexate (7OH-MTX) Synthesis and Characterization S3

UPLC-MS Analysis of MTX and its Metabolites S3

Stability and Drug Release Experiments in Cell Culture Medium and Rat Plasma S4

Didoceylmethotrexate (ddMTX) Synthesis and Characterization

100 mg of MTX and 71.5 mg of cesium carbonate and 120 µL of dodecyl bromide (corresponding to 125 mg; d = 1.04) were dissolved in 6 ml dimethylsulfoxide (DMSO) and left to react under stirring for 24 h. The molar ratio between methotrexate (MTX), cesium carbonate and dodecyl bromide is 1:1:2. ddMTX was precipitated from reaction mixture by adding 5 mL water and extracted with 3 mL chloroform for 5 times. The organic phase was reduced to 6 mL under nitrogen steam, and DMSO is removed by extracting with 5 mL sodium chloride saturated solution for 3 times. The organic phase was then evaporated under nitrogen steam and the residue was purified through Silica column by employing a chloroform/methanol gradient.

The obtained product was characterized through High Performance Liquid Chromatography (HPLC). Analyses were performed with a LC10 HPLC UV system (Shimadzu, Tokyo, Japan), linked to a Class LC10 software for data analysis. Column was a Teknokroma Kromosil 100 Si 25 cm × 0.46 nm, UV wavelength was set at 302 nm, mobile phase was dichlorometane/methanol 95/5 at 1 mL/min. ^{1}H and ^{13}C NMR spectra were recorded on a Jeol ECZ-R 600, at 600 and 150 MHz, respectively, using SiMe4 as internal standard. The following abbreviations are used to designate peak multiplicity: s = singlet, d = doublet, t = triplet, m = multiplet, bs = broad singlet. ESI-MS spectra were recorded on a Micromass Quattro API micro (Waters Corporation, Milford, MA, USA) mass spectrometer. Data were processed using a MassLynx System (Waters).

^{1}H NMR (300 MHz, $CDCl_3$) δ 8.66 (s, 1H), 7.72 (d, J = 9 Hz, 2H), 6.88 (d, J = 6 HZ, 1H), 6.75 (d, J = 9 Hz, 2H), 5.37 (bs, 2H), 4.75 (bs, 3H), 4.15 (t, J = 6 Hz, 1H), 4.02 (t, J = 6Hz, 2H), 3.18 (s, 3H), 1.70-1.50 (m, 2H), 1.40-1.15 (m, 40H), 0.88 (t, J = 6 Hz, 6H). ESI-MS $[M+H]^+$: m/z 792. HPLC UV: R_t = 8.5 min.

Lipophilic Linker *N*-Octadecil-3-Maleimido-Benzamide (ST-MBS) Synthesis and Characterization

4.5 mg stearylamine and 5 mg of 3-maleimidobenzoic acid *N*-hydroxysuccinimide ester (MBS) were dissolved in 1 mL of $CHCl_3$ and 15 µL di triethylamine and kept reacting at 40 °C for 4 h. The chloroform phase was extracted twice with 1% NaCl and twice with distilled water to remove water-soluble by-products. $MgSO_4$ was added to the chloroform phase and filtered. The mixture was dried from chloroform using a rotor-evaporator.

The reaction was assessed through Thin Layer Chromatography (TLC—acetic acid/chloroform/methanol 1/89/10). The product was characterized through HPLC. Analyses were performed with a YL9100 HPLC system equipped with a YL9110 quaternary pump, a YL9101 vacuum degasser and a YL9160 PDA detector, linked to YL-Clarity software for data analysis (Young Lin, Hogye-dong, Anyang, Korea). Column was a Teknocroma C18 mediterranea Sea 25 × 0.46 cm, PDA wavelengths were set at 220–290 nm, eluent flow was 1 mL/min, gradient was 0 min: 100% water, 20 min: 100% acetonitrile, 30 min: 100% acetonitrile, 35 min: 100% water. ^{1}H and ^{13}C NMR spectra were recorded on a Jeol ECZ-R 600, at 600 and 150 MHz, respectively, using SiMe4 as internal standard. The following abbreviations are used to designate peak multiplicity: s = singlet, d = doublet, t = triplet, m = multiplet, bs = broad singlet. ESI-MS spectra were recorded on a Micromass Quattro API micro (Waters Corporation, Milford, MA, USA) mass spectrometer. Data were processed using a MassLynx System (Waters).

TLC: ST-MBS R_f 0.88, MBS R_f 0.75, stearylamine R_f 0.28. ^{1}H NMR (600 MHz, $CDCl_3$) δ 7.76-7.74 (m, 1H), 7.73-7.71 (m, 1H), 7.54-7.52 (m, 1H), 7.49-7.47 (m,1H), 6.87 (s, 2H), 6.19-6.15 (m, 1H), 3.45-3.40 (m, 2H), 1.26-1.22 (m, 32H), 0.86 (t, J = 6 Hz, 3H). ESI-MS $[M+H]^+$: m/z 468. HPLC PDA: R_t = 25.6 min.

PEGylated Linker Stearyl-PEG-Maleimide (ST-PEG-MBS) Synthesis and Characterization

In the first step, 10 mg stearic acid (140 mM), 1.25 mg N-hydroxysuccinimide (NHS—43 mM), 1 mg 1-ethyl-3-(3-dimethylaminopropyl)carbodiimide (EDC—26 mM) were dissolved in 0.25 mL dimethylformammide (DMF) anhydrous and kept reacting overnight at 45 °C. The reaction mixture was diluted with 1 mL chloroform and washed with 4 mL 0.1 M HCl to eliminate water soluble compounds. The organic phase was treated with $MgSO_4$ and dried under nitrogen steam and checked through TLC (0.5% triethylamine in chloroform): being in excess of stearic acid, it was a mixture of the reagent and of activated stearic acid (36%). In a second step, 6.1 mg of first step reaction product (corresponding to 2.2 mg activated stearic acid—12 mM), 2 mg MBS (12 mM), 12 mg diamino-PEG (12 mM), 12 µL triethylamine (237 mM) were dissolved in 0.5 mL chloroform and kept reacting overnight at 45 °C. The reaction mixture was dried under nitrogen steam; the residue was dissolved in 400 µL ethanol, and 1.6 mL 1 M HCl was added to precipitate un-reacted stearic acid from the first step reaction. The obtained solution underwent size exclusion through a Sephadex G10 column to eliminate compounds with M_w < 500. ST-PEG-MBS was purified from un-reacted diamino-PEG through Dowex 50WX8-200 resin. The purified solution was freeze dried. The obtained conjugate (6.4 mg) was checked through TLC (acetic acid/chloroform/methanol 1/84/15) and characterized by ^{1}H-NMR (300 MHz ^{1}H were recorded on a Bruker 300 Avance instrument at 25 °C). The product was characterized through HPLC. Analyses were performed with a YL9100 HPLC system equipped with a YL9110 quaternary pump, a YL9101 vacuum degasser and a YL9160 PDA detector, linked to YL-Clarity software for data analysis (Young Lin, Hogye-dong, Anyang, Korea), and, alternatively, with a LC10 HPLC UV system (Shimadzu, Tokyo, Japan) equipped with a ELSD detector, linked to a Class LC10 software for data analysis. Column was a Teknocroma C18 mediterranea Sea 25 × 0.46 cm, PDA wavelengths were set at 220–290 nm, eluent flow was 1 mL/min, gradient was 0 min: 100% water, 20 min: 100% acetonitrile, 30 min: 100% acetonitrile, 35 min: 100% water.

TLC (0.5% triethylamine in chloroform) stearic acid R_f 0.2, NHS activated stearic acid R_f 0.46. TLC (acetic acid/chloroform/methanol 1/84/15) ST-PEG-MBS R_f 0.29, stearic acid R_f 0.9, MBS R_f 0.9, diamino-PEG R_f 0.02. HPLC-PDA. ST-PEG-MBS R_t = 14.6 min. HPLC-ELSD ST-PEG-MBS R_t = 15.0.

^{1}H NMR. The maleimide protons were detected at 6.5 ppm, and the stearic moiety could be easily evidenced at high field (1.2 ppm multiplet for the alkylic chain and 0.9 ppm for the methyl moiety). Both maleimide and stearic moiety were linked to diamino-PEG in 1:1 molar ratio.

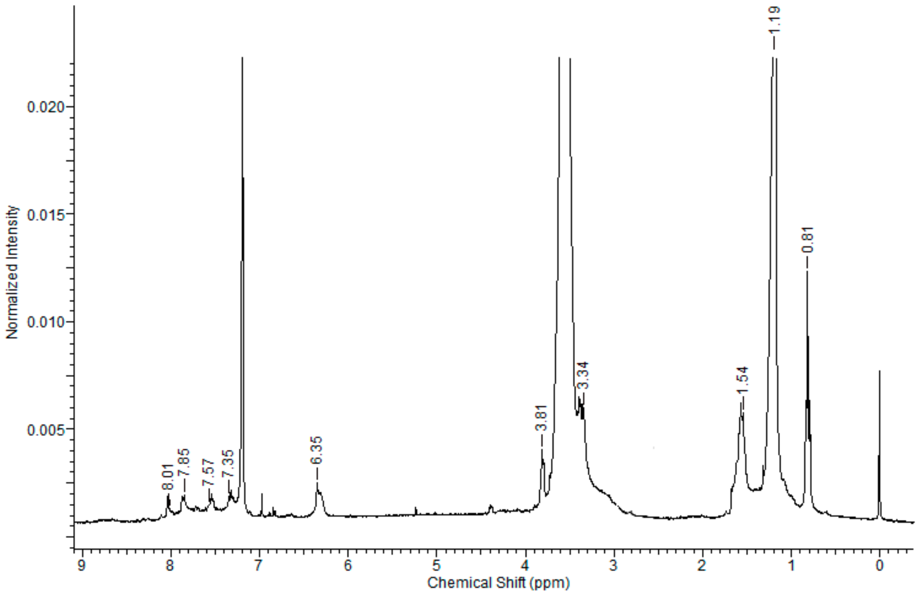

Figure S1. ^{1}H-NMR spectrum of ST-PEG-MBS.

7-Hydroxymethotrexate (7OH-MTX) Synthesis and Characterization

5 mg MTX were dissolved in 33 mL 0.133 M Sorensen buffer brought at pH = 7.8 with 5 M sodium hydroxide. 10 mL enzyme solution (corresponding to 112 mg enzyme) were slowly unfrozen in ice bath and then added to the MTX solution. The mixture was kept at 37 °C for 2 h. Afterwards the pH was brought to 8.4 with 0.5 M sodium hydroxide and kept at 40 °C overnight. The mixture was centrifuged at 5000 rpm (Rotofix 32A centrifuge, Hettich, Tuttlingen, Germany) and the supernatant was isolated, heated until 70 °C and centrifuged. Complete deproteinization was obtained with Amicon Centriflo (Millipore, Burlington, MS, USA) equipped with 50.000 Da dialysis membrane. The dialysed solution was brought to pH = 4.0 with acetic acid and kept at 4 °C overnight in order to obtain the complete precipitation of 7OH-MTX. The obtained suspension was centrifuged at 25,000 rpm (Allegra® 64R centrifuge, Beckmann Coulter, Paolo Alto, CA, USA), supernatant was discarded and the precipitate was dried under vacuum. Nearly 2 mg 7OH-MTX were obtained. Reaction product was assessed through PDA-HPLC described below: eluted fractions were collected and analyzed with mass spectroscopy (Micromass Quattro microTM API, Waters Corporation, Milford, MA, USA).

HPLC PDA: MTX R_t = 16.5 min, 7OH-MTX R_t = 20.2 min. PDA spectra: MTX main peaks 225 nm, 260 nm, 305 nm, 370 nm; 7OH-MTX main peaks 230 nm, 305 nm [25]. ESI-MS $[M-H]^-$: *m*/*z* 469.

UPLC-MS Analysis of MTX and its Metabolites

MTX, 7OH-MTX and urine samples underwent also UPLC-MS analysis owing to the following conditions. UPLC-MS analysis was performed with an Acquity UPLC (Waters Corporation, Milford MA, USA) equipped with BSM, SM, CM and PDA detectors. The analytical column was a Zorbax Eclipse XDB-C18 150 × 4.6 mm. The mobile phase consisted of methanol and ammonium acetate buffer pH = 6.0 (30/70). UPLC retention time (R_t) was obtained at flow of 0.4 mL/min, and the column effluent was monitored using Micromass Quattro microTM API Esci multi-mode ionization enabled as detector. The MS conditions were: purging gas (nitrogen) heated at 350 °C at a flow rate of 800 L/h; nebulizer gas (nitrogen) at 80 L/h; capillary voltage in negative mode at 3000 V; fragmentor voltage 20 V.

UPLC-MS chromatogram of standard MTX and 7OH-MTX and a sample of urine: MTX R_t = 3.6 ESI-MS $[M-H]^-$: *m*/*z* 453; 7OH-MTX R_t = 3.8 ESI-MS $[M-H]^-$: *m*/*z* 469.

Stability and Drug Release Experiments in Cell Culture Medium and Rat Plasma

ddMTX loaded behenic acid SLN, purified through size exclusion (Sepharose CL-4B), were diluted 50 folds in cell culture medium or in rat plasma and kept under magnetic stirring. At scheduled times, 50 µL of the release medium were centrifuged at 25,000 rpm (Allegra® 64R centrifuge, Beckmann Coulter, Paolo Alto, CA, USA) and the supernatant was diluted with 200 µL water, before being derivatized for fluorescence HPLC detection. Particle size was measured before and at the end of the experiment in cell culture medium or in rat plasma.

Table S1. Particle size of ddMTX loaded behenic acid SLN in cell culture medium and rat plasma.

ddMTX Loaded Behenic Acid SLN	Particle Size	
**Pure SLN**	Mean size (nm)	389.6 ± 45
	Polydispersity	0.145
**In cell culture medium**	Mean size (nm)	397.5 ± 30.4
	Polydispersity	0.226
**In rat plasma**	Mean size (nm)	296.1 ± 44.6
	Polydispersity	0.302

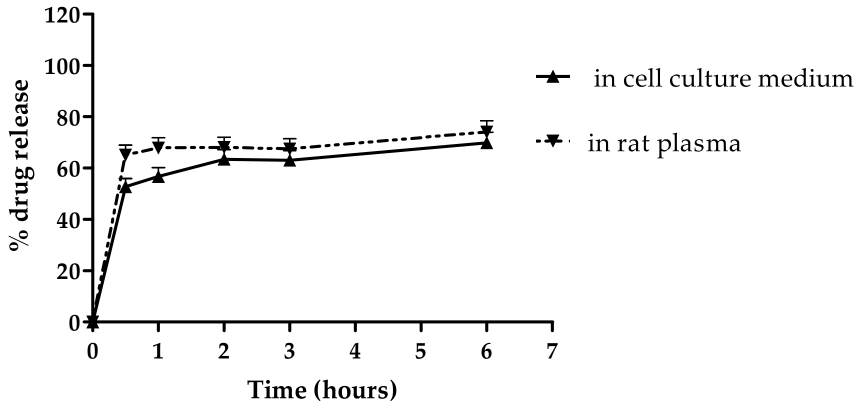

Figure S2. Drug release from ddMTX loaded behenic acid SLN.
